# Orbital Subperiosteal Abscess Drainage: Transorbital vs. Endonasal Approach †

**DOI:** 10.3390/jcm13237011

**Published:** 2024-11-21

**Authors:** Shreya Luthra, Andrea L. Kossler, Benjamin P. Erickson, Natalie A. Homer

**Affiliations:** 1 Faculty of Medicine, University of British Columbia, Vancouver, BC V6T 1Z4, Canada; sluthra1@student.ubc.ca; 2 Division of Ophthalmic Plastic and Reconstructive Surgery, Department of Ophthalmology, Byers Eye Institute, Stanford University, Palo Alto, CA 94303, USA; 3 Department of Ophthalmology, Kaiser Permanente, Santa Clara, CA 95051, USA; 4 Department of Ophthalmology, Casey Eye Institute, Oregon Health and Science University, Portland, OR 97239, USA

**Keywords:** orbital subperiosteal abscess, endoscopic drainage, external drainage, orbitotomy

## Abstract

**Background**: This study aims to compare the surgical outcomes of transorbital versus endonasal endoscopic approaches for orbital subperiosteal abscess drainage. **Methods**: A retrospective review was conducted at a single institution of patients who underwent orbital subperiosteal abscess drainage from November 2009 to April 2023. **Results**: Of 64 patients, 44 (68.8%) underwent abscess drainage via an orbital approach, while 20 (31.3%) underwent endonasal endoscopic drainage. No significant difference in operative time or visual acuity improvement was found between the two groups. Abscess drainage via orbitotomy was associated with a longer total (average 8.1 days) and postoperative (average 7.3 days) hospitalization time compared to the endoscopic group (average 5.4 days and 4.2 days, respectively), though this difference was not statistically significant (*p* = 0.197, 0.136, respectively). For medial orbital abscesses, the average length of total and postoperative hospitalization was greater after orbitotomy (*p* = 0.028 and 0.019, respectively). At discharge, patients who underwent orbitotomy more commonly reported periorbital swelling (*p* = 0.0003), while postoperative pain was more common in the endoscopic drainage group (*p* = 0.009). Reoperation rate was higher after orbitotomy (34.1%) compared to the endoscopic drainage group (15.0%), though this was not statistically significant (*p* = 0.115). **Conclusions**: Transorbital and endoscopic surgical approaches for orbital abscess drainage have similar surgical outcomes, with no statistically significant differences noted in terms of visual acuity change or reoperation rate. Longer hospital stays were found for patients with medial orbital abscesses drained via orbitotomy.

## 1. Introduction

Subperiosteal orbital abscesses (SPOA) are observed in approximately 15% of cases of infectious sinusitis [1]. These abscesses often present with periorbital edema, pain, chemosis, diplopia, ophthalmoplegia, proptosis and globe malposition [2]. Without timely and effective treatment, SPOA and concomitant sinusitis may lead to vision loss, endophthalmitis, cavernous sinus thrombosis, meningitis, intracranial abscesses and even death [3]. While some orbital abscesses respond to medical treatment, surgical intervention may be required if there is a progression of orbital signs and symptoms despite medical therapy. Orbital abscesses can be classified based on Chandler’s original classification system, which divides orbital cellulitis into five stages [4]. Stage I (preseptal cellulitis) indicates eyelid inflammation in the absence of edema of orbital contents. Stage II (postseptal cellulitis) indicates inflammation of the orbital contents without discreet abscess formation. Stage III indicates the presence of a SPOA, and Stage IV indicates an orbital abscess within the orbital tissues [4,5]. This classification system recommends surgical intervention for Stage III and IV patients [2], with goals to reduce the pressure on the orbit, drain purulent cystic contents, and obtain microbial culture [5]. Garcia and Harris [6] advised expectant management except in cases with the following criteria: patients aged greater than 9 years, the presence of frontal sinusitis, a large or non-medial SPOA location, suspicion of anaerobic subperiosteal infection (e.g., presence of gas within the abscess space on computed tomography (CT) imaging), recurrence of SPOA after previous drainage, evidence of chronic sinusitis (e.g., nasal polyps), acute optic nerve or retinal compromise, or infection of dental origin. Additional guidelines proposed for clinical criteria warranting abscess drainage include ophthalmoplegia, 20/60 (or worse) visual acuity on initial evaluation, or lack of improvement within 48 h despite medical treatment [7,8]. Abscess width is also considered in the decision for surgical drainage [9,10]. Oxford and McClay [9] suggested the criteria of normal vision, no ophthalmoplegia, proptosis < 5 mm, intraocular pressure <20 mmHg, and abscess width < 4 mm as appropriate parameters for medical therapy. 

Surgical abscess drainage may be pursued by an endonasal endoscopic approach or a transorbital approach, via a transcaruncular or transcutaneous incision. Historically, the drainage of a medial abscess was accomplished via a Lynch-Howarth or Modified Lynch incision. In this technique, dissection is conducted through the subperiosteal space and provides a direct route to the abscess with good visualization. However, this technique carries the risk of medial canthal tendon or lacrimal canaliculi damage, facial scarring, webbing, delayed healing, and suture abscess [5,11,12,13,14]. In 1990, Shorr et al. [14] proposed a less-invasive transcaruncular approach to achieve greater inferior and medial orbital wall exposure and to reduce postoperative scarring. This less-invasive method allows greater inferior and medial orbital wall exposure with reduced postoperative scarring, making it a more widely utilized technique today.

Advantages of the orbital approach include the ability for preservation of an intact medial orbital wall and the option for drain placement. Drains can be placed during transorbital orbitotomy to allow for postoperative evacuation of purulent and serous fluid, thus relieving pressure within the orbital cavity and decreasing infection burden. Currently, no large studies have compared the surgical outcomes of patients who underwent abscess evacuation via orbitotomy with and without drain placement.

Transnasal endoscopic abscess drainage is a minimally invasive approach offering several advantages, including the avoidance of facial scars and decreased morbidity [2]. Other specialties have used indirect endoscopic approaches as a successful avenue for the treatment of skull base lesions [15,16]. This technique can be easily performed at the time of functional endoscopic sinus surgery. However, the endoscopic approach may be limited by decreased visibility, raising the risk of medial rectus muscle or optic nerve damage, and incomplete drainage requiring revision surgery [2,3,13]. Additionally, this approach requires lamina papyracea violation and limits the ability to place a drain. 

While there exists a considerable body of literature describing endonasal and transorbital surgical approaches to orbital abscess drainage, there have been no large studies conducted to compare and assess the safety, efficacy, and appropriateness of each method. The purpose of this study was to compare patient outcomes following transorbital and endonasal approaches to orbital abscess drainage to guide optimal patient care. 

## 2. Materials and Methods 

A retrospective review was performed at a single institution from November 2009 to April 2023 of all patients who underwent orbital abscess drainage from an endoscopic approach or external orbital approach. All patients, regardless of the surgical approach to orbital abscess drainage, underwent simultaneous functional endoscopic sinus surgery for sinusitis management. Patient demographics, clinical presentation, abscess characteristics, ophthalmologic manifestations, radiology findings, and medical management (i.e., systemic antibiotic and steroid use) were collected. CT scans were performed on all patients to determine abscess size and location. The transcaruncular approach was performed in a similar manner to that initially described by Shorr et al., which involves an incision through the lacrimal caruncle and medial conjunctiva, followed by blunt dissection to the level of the medial orbital wall and entry into the medial orbital subperiosteal space [14]. The endonasal endoscopic approach involves making an isolated entry into the lamina papyracea in the area of the abscess noted on preoperative imaging, followed by gentle globe ballottement allowing for the expulsion of purulent material from the orbital subperiosteal space. The effectiveness of each approach was determined by comparing the following outcome measures: patients’ total length of hospitalization, the length of postoperative hospitalization, change in visual acuity pre vs. post operation, operative time, surgical complications, and the need for revision surgery. Visual acuity was collected at the time of presentation to hospital, prior to hospital discharge, and upon outpatient follow-up. Postoperative symptoms were obtained from a review of the patient’s hospital record, using information from the subjective and objective portions of the ophthalmology follow-up documentation, corroborated by clinical photographs when available. Subgroup analysis was conducted for patients undergoing external orbitotomy drainage with and without drain placement. Additionally, subgroup analysis of patients with medially located orbital subperiosteal abscesses was performed. Patients with dental caries, periapical abscesses, and odontogenic infections were excluded. Statistical analysis was performed using Chi-squared and *t*-tests, with *p*-values < 0.05 considered significant. This study adhered to the tenets of the Declaration of Helsinki, and IRB approval was obtained. The collection and evaluation of protected patient health information were HIPAA-compliant.

## 3. Results 

A total of 64 patients, including 44 males (68.8%) and 20 females (31.3%), with a median age of 23 years (range 2–84 years), were included (Table 1). Overall, 62 patients exhibited unilateral abscesses, while 2 had bilateral orbital abscesses. Common clinical presentations included periorbital edema, erythema, ophthalmoplegia, chemosis, proptosis, fever, significant ptosis, and vision changes. Acute microbial sinusitis (90.6%) was the most common etiology (Table 1). All patients were treated with intravenous antimicrobial therapy, most commonly Vancomycin or Piperacillin-Tazobactam. The most common pathogens were *Streptococcus anginosus* and *Methicillin-resistant Staphylococcus aureus.* A total of 44 patients (68.8%) underwent abscess drainage via an orbital approach at the time of endoscopic sinus surgery, while 20 patients (31.3%) underwent endonasal endoscopic abscess drainage. Visual acuity and symptoms at outpatient follow-up were performed on average 29.2 days after surgery (range 2–136 days).

Approaches to external drainage were categorized by incision location, including upper eyelid crease (36.4%), transcaruncular (31.8%), subbrow (22.7%), and Lynch incisions (9.1%). Fifty percent (n = 22) of patients who underwent orbitotomy had a drain (Jackson-Pratt or Penrose) placed at the time of surgery.

Orbitotomy and endoscopic treatment groups were compared. No statistically significant differences were observed in terms of gender, abscess laterality, visual acuity at presentation, presenting symptoms, antibiotic selection, or perioperative steroid use between the treatment groups. A statistically significant difference was observed for mean age at presentation between the orbitotomy and endoscopic drainage groups (28.1 years and 16.2 years, respectively, *p* = 0.036). Days from symptom onset to hospital presentation, days from symptom onset to surgical drainage, and duration from presentation to drainage were similar between the two groups (Table 1). Analysis of the abscess location and depth revealed that longer anterior-to-posterior abscesses (*p* = 0.005) and superiorly located orbital abscesses (*p* = 0.042) were more commonly drained via orbitotomy, while medial abscesses were more often drained endoscopically (*p* = 0.0003). The average operative time did not differ significantly between the orbitotomy (145.3 min) and endoscopic (117.4 min) groups (*p* = 0.181). No significant differences were noted in abscess pathogens. Perioperative steroid treatment was administered to 70.5% of externally drained patients and 75.0% of endoscopically drained patients (*p* = 0.710).

Abscess drainage via orbitotomy was associated with a longer average total hospitalization length (8.1 days, range 2 to 44 days) and postoperative hospitalization (7.3 days, range 0 to 43 days) compared to the endoscopic group (5.4 days and 4.2 days, respectively), though this difference was not statistically significant (*p* = 0.197, 0.136, respectively) (Table 2). At discharge, patients who underwent orbitotomy were more likely to have periorbital swelling (45.5% vs. 0%, *p* = 0.0003), while postoperative pain was more common in endoscopically treated patients (15.0% vs. 0%, *p* = 0.009) (Table 3). The proportion of cases requiring reoperation was higher after orbitotomy (34.1%) compared to the endoscopic drainage group (15.0%), though this was not statistically significant (*p* = 0.115). No significant differences were observed in visual acuity improvement from admission to discharge, or visual acuity at discharge or outpatient follow-up, between the two groups. 

Of the 44 patients who underwent external orbitotomy for abscess drainage, 22 (50%) had a drain placed at the time of surgery (Table 4). No statistically significant differences in sex, age at diagnosis, abscess laterality, visual acuity at presentation, antibiotic selection, or perioperative steroid use were present between the drain vs. no-drain groups. Drains were more often placed for patients with lateral (*p* = 0.01) and superior (*p* = 0.001) abscesses. Abscess size did not correlate with decision for drain placement. Days from symptom onset to hospital presentation, days from symptom onset to surgical drainage, and duration from presentation to drainage were similar for the drain (average 4.6, 6.0, 1.4 days, respectively) and no-drain (average 7.5, 8.7, 1.2 days, respectively) groups. The mean operative time was not statistically different between patients with (159.8 min) and without (127.5 min) drain placement (*p* = 0.21). The percentage of patients requiring reoperation for abscess recurrence was higher after drain placement (40.9%) compared to the no-drain group (27.3%), though this was not statistically significant (*p* = 0.345). The length of total and postoperative hospitalization was not significantly different between the two groups (drain: 8.2, 7.0, respectively; no-drain: 8.1, 7.7, respectively). No significant differences were observed in postoperative symptoms, visual acuity improvement from admission to discharge, or visual acuity at discharge or outpatient follow-up, between the two groups. 

Of the 64 total patients included in the study, 36 (56.3%) had medial orbital abscesses and were additionally assessed as a subgroup. Eighteen (50%) of the patients with medial abscesses underwent external orbitotomy (Table 5). No statistically significant differences in sex, age at diagnosis, abscess laterality, visual acuity at presentation, antibiotic selection, or perioperative steroid use were present when comparing patients with medial abscesses treated with external orbitotomy compared to those treated endoscopically. Medial abscess size was not significantly different between the two surgical drainage approaches. The number of days from symptom onset to hospital presentation, days from symptom onset to surgical drainage, and the duration from presentation to drainage were also compared between the two groups and were not found to be statistically significant (external: average 4.8, 6.6, 1.7 days, respectively; endoscopic: average 4.3, 5.8, 1.6 days, respectively). The mean operative time was similar between the external orbitotomy (164.0 min) and endoscopic (111.2 min) groups (*p* = 0.061). The percentage of patients requiring reoperation and repeat drainage was higher after external orbitotomy (44.4%) compared to the endoscopic group (11.1%), though this did not reach statistical significance (*p* = 0.06). The length of total and postoperative hospitalization was greater when medial abscesses were treated with external orbitotomy (12.6, 11.7 days, respectively) compared to endoscopic drainage (5.3, 4.2 days, respectively) (*p* = 0.028, 0.019, respectively). At the time of discharge, patients who underwent orbitotomy were more likely to have periorbital swelling (27.8% vs. 0%, *p* = 0.046). No significant differences were observed in visual acuity improvement from admission to discharge, or visual acuity at discharge or outpatient follow-up, between the two groups.

## 4. Discussion 

Orbital subperiosteal abscesses are a common complication of infectious sinusitis and may necessitate surgical drainage. Both transorbital and endoscopic approaches have been well established. Endoscopic endonasal drainage can be easily performed by either the oculoplastic or otolaryngology provider at the time of functional endoscopic sinus surgery, while a transorbital approach may be utilized to avoid disruption of the lamina papyracea and allow for drain placement. Only limited small studies exist comparing orbital abscess surgical drainage techniques [3,17,18,19]. At the current institution, the Garcia–Harris criteria are utilized to guide the decision of surgical intervention [6]; however, these criteria do not dictate which surgical approach is pursued. The review herein found the surgical approach to be influenced by age, abscess location, and abscess anterior-to-posterior length, with older patients with superior abscesses of longer length more commonly undergoing orbitotomy. 

Increased orbital abscess size has previously been correlated with higher risk of surgical failure [17]. Thus, external or combined abscess drainage has been advised for larger abscesses to allow for direct and comprehensive evacuation [17]. In the present study, abscesses with longer anterior-to-posterior dimension were more commonly drained externally. Furthermore, abscess location logically influences orbital abscess drainage approach, with external orbitotomy serving as a more appropriate approach for superior or laterally extending abscesses given further proximity from the ethmoidal sinuses [3,11,17,20,21,22]. Conversely, the transnasal endoscopic approach is more feasible for complete medial subperiosteal abscess drainage [3,11,21,22,23]. While inferior orbital abscesses were not common in our study, prior literature has suggested an increased risk for incomplete drainage with endoscopic approaches [23]. Superior orbital abscesses can be successfully drained from an external or combined approach, which may include frontal sinusotomy [24]. Contrarily, other studies have noted that the transnasal endoscopic approach is sufficient in treating orbital abscesses regardless of location [22,25]. The present study acknowledges the relevance of abscess location as a significant factor in the selection of surgical technique. 

In limiting hospitalization time, the endoscopic approach has historically demonstrated superiority as it does not require an external incision, produces less postoperative edema, and ensures thorough sinus debridement in the area of the abscess to allow access [11,12,19]. Page and Wiatrak [18] found a trend towards longer mean total hospital stay and postoperative stay after the transorbital approach (7.4 days and 6.3 days, respectively) when compared to endoscopic drainage (6.0 days and 4.3 days, respectively). However, this difference did not reach statistical significance in their small study of fourteen patients. Subsequent small studies have similarly reported a longer hospitalization when an orbital or combined approach is utilized [11,12,19]. Chorney et al. [26] conducted a pediatric study involving sixteen children, comparing the length of stay between eleven children who underwent combined external and endoscopic drainage and five children who underwent external drainage alone. The study revealed no significant difference in the median length of stay between patients treated with the combined approach and those treated with external drainage alone. The present study found a trend toward reduced total and postoperative hospitalization with endoscopic abscess drainage that did not reach significance when including abscesses in all locations. Upon subgroup analysis of medial abscesses only, the length of total and postoperative hospitalization was significantly longer for those drained via external orbitotomy compared to endoscopic drainage. This may suggest that an endoscopic approach, particularly in the case of medial SPOA in which the two surgical techniques are often be equally feasible, could be beneficial in reducing healthcare resource demands. Further large prospective studies in which patients are randomized into one of the two surgical approaches, ensuring parity within the treatment groups, would be necessary to confirm these results.

Several studies have analyzed the rates of reoperation and surgical failure, defined as abscess persistence or recurrence, in this pathology, as suboptimal drainage may lead to devastating complications [3,17,18,19]. Arjmand et al. [19] reported incomplete drainage in 3 out of 22 cases, including 1 in which cerebritis developed after endoscopic drainage. Another child presented with a frontal empyema after receiving initial endoscopic treatment at another hospital. Cheng et al. [13] found a statistically significant increase in adverse events for endoscopically treated patients who underwent delayed surgery (*p* < 0.0001), with an unplanned reoperation rate of 5.3%. Other small studies have compared reoperation rates between the two techniques. Page and Wiatrak [18] had one patient out of seven initially treated endoscopically requiring reoperation. In a small study involving 11 pediatric patients, Pelton et al. [27] reported a reoperation rate of 14.3% after combined endoscopic and orbital drainage, compared to a 25% reoperation rate after orbital drainage alone. Rahbar et al. [20] reported the need for reoperation in 2 out of 11 endoscopically drained patients within 24 to 48 h after surgery. Additionally, Rubin et al. [17] found that the percentage of surgical failures requiring additional drainage was almost twice as high after endoscopic drainage (failure rate: 25%) than after external drainage (failure rate: 14.3%); however, this difference was not significant. Previous studies have highlighted endoscopic drainage to be associated with increased rates of surgical failure particularly when SPOA are inferiorly located or have a greater length and width [18]. Our study with notably higher patient numbers than those previously reported revealed reoperation rates were higher after external orbitotomy compared to the endoscopic group in all abscesses (34.1% vs. 15.0%) and in the medial abscess subgroup (44.4% vs. 11.1%). However, these differences did not achieve statistical significance in either group (*p* = 0.115 and 0.06, respectively). Thus, the data herein do not support a particular approach to being superior in achieving complete SPOA abscess drainage. 

Few studies have analyzed the impact of drain placement during external orbitotomy on patient outcomes. Gupta et al. [28] conducted a comparative analysis of patient outcomes following anterior orbitotomy, examining the impact of drain placement versus no drain placement during surgery. This study found that postoperative proptosis (*p* = 0.04), chemosis (*p* = 0.04), and ocular motility (*p* = 0.04) were significantly better in the group that had a drain placed compared to those who did not. However, long-term outcomes and complications at 30 days were comparable in both groups. In the present study, abscess size did not impact surgeon decisions regarding drain placement, whereas abscess location, particularly patients with lateral and superior abscesses, were significantly more likely to have a drain placed at the time of surgery. Drain placement did not have an impact on surgical or patient outcomes, and no significant differences were found between the drain vs. no-drain groups for total or postoperative hospitalization, operative time, reoperation rates, postoperative symptoms, or visual acuity. Therefore, the unique ability of drain placement with the orbital approach does not appear to positively impact post-surgical outcomes. 

External drainage has previously been associated with significant inflammation and greater than average bleeding compared to endoscopic sinus surgery [19]. Endoscopic drainage has been relatively contraindicated for specific cases, including patients with bleeding disorders that may compromise intraoperative visibility, anatomic variations that make approach to the medial orbit difficult, and concerns for loss of visual acuity as the endoscopic procedure can take more time than the external approach [12,17]. Previous studies have compared the impact of surgical approach on postoperative symptoms, visual acuity, and vision loss. Migirov et al. [11] found that the mean duration of postoperative healing was significantly shorter in the endoscopic drainage group (4.2 ± 1.9 days) compared to the external drainage group (8.6 ± 4.2 days) (*p* = 0.005). Further, postoperative sequelae were frequently found in children treated via the external approach including scarring, stitch abscesses, delayed healing, unresolved diplopia, and recurrent periorbital cellulitis, with or without subperiosteal abscess. Notably, the postoperative course was uneventful in all children who had been treated endoscopically—aside from non-clinically significant intranasal synechiae [11]. Page and Wiatrak [18] compared visual acuity pre- and postoperatively and found that no patient suffered permanent vision loss regardless of the surgical approach taken. However, when comparing postoperative symptoms, patients treated with transorbital drainage were more likely to have edema when compared to patients treated endoscopically. These findings are supported by the present study which found a greater risk of periorbital edema in patients who underwent external orbitotomy and greater postoperative pain in patients treated endoscopically. Notably, increased periorbital edema upon discharge following external orbitotomy was found in both the subgroup and large cohort analysis, which further supports this distinctive postoperative experience and may aid in preoperative patient counseling and expectation setting. No major complications or recurrence were observed in either the transorbital or the endoscopic endonasal group in our study. Additionally, no statistically significant differences were noted in ipsilateral visual acuity at admission, discharge, or follow-up (up to 12 weeks post operation) between the two groups. 

This study is limited by its retrospective nature, single institution setting, and fairly small patient number (albeit the largest comparison study for these surgical techniques in this pathology to date). Lack of standardization of surgical practices amongst practitioners at the study institution both serves as a limitation in decreasing treatment group cohesiveness, whilst favorably increasing the generalizability of study results. Differences in abscess anterior-to-posterior depth between the two groups may suggest disparity in infection severity. However, patients in both groups compared similarly in presenting visual acuity change and periorbital symptoms. Given the retrospective nature of this review, this study relied on the documentation provided by the ophthalmology team which was presumed to be thorough; however, there remains the possibility that some pre- and postoperative symptoms and signs were inadvertently missed or not recorded. Future prospective studies could utilize a standardized questionnaire to evaluate these symptoms. Varying average age between treatment groups also may influence outcome variables, such as patient length of stay and symptoms at the time of discharge. However, the age-specific technique preferences compared similarly with those previously reported, [1,11,20,29] and can likely only be overcome in a randomized controlled study. Finally, the maximum follow-up duration of 12 weeks post-surgery may not fully capture long-term complications such as an unaesthetic scar or delayed mucocele and synechiae formation. These authors call for future large prospective multi-institutional studies for further confirmation of study findings.

## 5. Conclusions

Orbital subperiosteal abscesses may be successfully drained via external orbital or endonasal endoscopic approaches. Factors influencing drainage technique choice include patient age and abscess characteristics of location and length. The orbital abscess surgical approach, with or without drain placement, does not significantly influence vision outcomes, complications or reoperation rates. There is a trend towards longer hospital stays in those who undergo orbitotomy that reached statistical significance when solely evaluating patients with medial orbital abscesses. These findings suggest increased consideration for endoscopic orbital abscess drainage to reduce the hospital resource burden, which should be confirmed in larger prospective studies. 

## Figures and Tables

**Table 1:** Patient demographics.

	Orbitotomy	Endoscopic	p-Value
**Total**	44 (68.8%)	20 (31.3%)	
***Patient Characteristics***			
Mean age at diagnosis, y	28.1	16.2	0.036 *
Female sex	13 (29.6%)	7 (35.0%)	0.665
***Etiology***			
Sinusitis	39 (88.6%)	19 (95.0%)	0.422
Trauma	3 (6.8%)	1 (5.0%)	0.782
Postoperative	2 (4.5%)	0	0.336
***Abscess Laterality***			
Right	20 (45.5%)	12 (60.0%)	0.284
Left	23 (52.3%)	7 (35.0%)	0.203
Both	1 (2.3%)	1 (5.0%)	0.564
***Abscess Location***			
Medial	18 (40.9%)	18 (90.0%)	0.0003 *
Superior	12 (27.3%)	1 (5.0%)	0.042 *
Lateral	9 (20.5%)	1 (5.0%)	0.117
Inferior	2 (4.5%)	0	0.336
Anterior	6 (13.6%)	0	0.235
***Mean Abscess Size (mm)***			
Transverse	19.9	18.8	0.679
Depth	13.8	7.3	0.005 *
***Days from***			
Symptom onset to diagnosis	6.1	4.1	0.223
Symptom onset to drainage	7.3	5.7	0.305
Diagnose to drainage	1.3	1.6	0.474
***Visual acuity* ** ^†^ ***(LogMAR, Snellen)***			
Mean—At presentation	0.73, 20/100	0.86, 20/140	0.702
Median—At presentation	0.18, 20/30	0.10, 20/25	0.814
***Abscess Pathogen***			
*Streptococcus anginosus*	13 (29.5%)	10 (50.0%)	0.116
MSSA ^‡^	6 (13.6%)	2 (10.0%)	0.689
MRSA ^§^	9 (20.5%)	2 (10.0%)	0.302
Other	16 (36.4%)	6 (30.0%)	0.620
***Steroid Treatment***	31 (70.5%)	15 (75.0%)	0.710

^†^ Visual acuity in ipsilateral eye. ^‡^ MSSA = *Methicillin-susceptible staphylococcus aureus*. ^§^ MRSA = *Methicillin-resistant Staphylococcus aureus*. * denotes *p*-value < 0.05.

**Table 2:** Outcome measures.

	Orbitotomy	Endoscopic	*p*-Value
***Hospital Stay (days)***			
Length of stay	8.1	5.4	0.197
Postoperative stay	7.3	4.2	0.136
***Operative time (mins)***			
Mean	145.3	117.4	0.181
Median	142.0	102.50	0.060
***Visual acuity*** ^†^ ***(LogMAR, Snellen)***			
Mean—At discharge	0.58, 20/80	0.86, 20/160	0.555
Mean—At follow up	0.46, 20/63	0.78, 20/125	0.501
Median—At discharge	0.14, 20/32	0.05, 20/20	0.789
Median—At follow up	0.10, 20/24	0.00, 20/20	0.779
***Reoperation***	15 (34.1%)	3 (15.0%)	0.115

^†^ *Visual acuity of affected eye*.

**Table 3:** Symptoms.

	Orbitotomy	Endoscopic	*p*-Value
***Presenting Symptoms***			
Swelling	40 (90.9%)	17 (85.0%)	0.487
Itchiness	7 (15.9)	2 (10.0%)	0.532
Erythema	8 (18.2%)	5 (25.0%)	0.534
Ophthalmoplegia	10 (22.7%)	2 (10.0%)	0.231
Pain	19 (43.2%)	8 (40.0%)	0.812
Chemosis	1 (2.3%)	2 (10.0%)	0.181
Proptosis	7 (15.9%)	4 (20.0%)	0.689
Fever	13 (29.5%)	10 (50.0%)	0.116
Vision Changes *	18 (40.9%)	5 (25.0%)	0.222
Discharge	7 (15.9%)	6 (30.0%)	0.197
***Discharge Symptoms***			
Swelling	20 (45.5%)	0	0.0003 *
Itchiness	2 (4.5%)	2 (10.0%)	0.402
Erythema	3 (6.8%)	1 (5.0%)	0.784
Ophthalmoplegia	3 (6.8%)	0	0.236
Pain	0	3 (15.0%)	0.009 *
Chemosis	3 (6.8%)	0	0.236
Proptosis	1 (2.3%)	0	0.498
Fever	0	0	
Vision changes ^†^	1 (2.3%)	0	0.498
Discharge	2 (4.5%)	2 (10.0%)	0.402

^†^ *Vision changes include diplopia, photophobia, and blurry vision*. * denotes *p*-value < 0.05

**Table 4:** Outcome measures: drain vs. no-drain.

	Drain	No-Drain	*p*-Value
**Total**	22 (50%)	22 (50%)	
***Abscess Location***			
Medial	9 (40.9%)	9 (40.9%)	1.000
Lateral	1 (4.6%)	8 (36.4%)	0.010 *
Superior	11 (50%)	1 (4.6%)	0.001 *
Inferior	0	2 (9.1%)	0.153
Anterior	1 (4.6%)	2 (9.1%)	0.555
***Hospital Stay (days)***			
Length of stay	8.2	8.1	0.950
Postoperative stay	7.0	7.7	0.792
***Operative time (mins)***	159.8	127.5	0.210
***Visual acuity*** ^†^ ***(LogMAR, Snellen)***			
At discharge	0.59, 20/78	0.58, 20/76	0.976
At follow up	0.53, 20/68	0.37, 20/47	0.670
***Reoperation***	9 (40.9%)	6 (27.3%)	0.345

^†^ *Visual acuity of the affected eye*. * denotes *p*-value < 0.05

**Table 5:** Outcome measures: medial abscesses.

	Orbitotomy	Endoscopic	*p*-Value
**Total**	18 (50%)	18 (50%)	
***Hospital Stay (days)***			
Length of stay	12.6	5.3	0.028 *
Postoperative stay	11.7	4.2	0.019 *
***Operative time (mins)***	164.0	111.2	0.061
***Visual acuity*** ^†^ ***(LogMAR, Snellen)***			
At discharge	0.80, 20/126	0.58, 20/76	0.630
At follow up	0.82, 20/132	0.49, 20/62	0.498
***Reoperation***	8 (44.4%)	2 (11.1%)	0.060
***Reoperation***	8 (44.4%)	2 (11.1%)	0.060

* denotes *p*-value < 0.05.

## Data Availability

There is a protected datasheet that is available.

## References

[1] Hassan E., Gendeh B.S., Husain S., Faizah M.Z. (2014). Orbital complications secondary to acute sinusitis– A 10 years retrospective review. East. J. Med..

[2] Sekhar V., Ao J., Iqbal I., Ooi E.H., Munn Z. (2019). Effectiveness of endoscopic versus external surgical approaches in the treatment of orbital complications of rhinosinusitis: A systematic review protocol. JBI Evid. Synth..

[3] Tanna N., Preciado D.A., Clary M.S., Choi S.S. (2008). Surgical Treatment of Subperiosteal Orbital Abscess. Arch. Otolaryngol. –Head Neck Surg..

[4] Chandler J.R., Langenbrunner D.J., Stevens E.R. (1970). The pathogenesis of orbital complications in acute sinusitis. Laryngoscope.

[5] Wang Y., Zhang J., Dong L., Jiang H., Song X. (2019). Orbital abscess treated by ultrasound-guided fine needle aspiration and catheter drainage. Medicine.

[6] Garcia G.H., Harris G.J. (2000). Criteria for nonsurgical management of subperiosteal abscess of the orbit22: Analysis of outcomes 1988–1998. Ophthalmology.

[7] Younis R.T., Lazar R.H., Bustillo A., Anand V.K. (2002). Orbital Infection as a Complication of Sinusitis: Are Diagnostic and Treatment Trends Changing?. Ear Nose Throat J..

[8] Harris G.J. (1994). Subperiosteal Abscess of the Orbit: Age as a Factor in the Bacteriology and Response to Treatment. Ophthalmology.

[9] Oxford L.E., McClay J. (2006). Medical and surgical management of subperiosteal orbital abscess secondary to acute sinusitis in children. Int. J. Pediatr. Otorhinolaryngol..

[10] Ryan J.T., Preciado D.A., Bauman N., Pena M., Bose S., Zalzal G.H., Choi S. (2009). Management of pediatric orbital cellulitis in patients with radiographic findings of subperiosteal abscess. Otolaryngol. –Head Neck Surg..

[11] Migirov L., Yakirevitch A., Bedrin L., Wolf M. (2009). Endoscopic sinus surgery for medial orbital subperiosteal abscess in children. J. Otolaryngol. Head Neck Surg..

[12] Bhargava D., Sankhla D., Ganesan A., Chand P. (2001). Endoscopic sinus surgery for orbital subperiosteal abscess secondary to sinusitis. Rhinology.

[13] Cheng J., Liu B., Farjat A.E., Jang D.W. (2017). Adverse Events in Endoscopic Sinus Surgery for Infectious Orbital Complications of Sinusitis: 30-Day NSQIP Pediatric Outcomes. Otolaryngol. Head Neck Surg..

[14] Timoney P.J., Sokol J.A., Hauck M.J., Lee H.B.H., Nunery W.R. (2012). Transcutaneous Medial Canthal Tendon Incision to the Medial Orbit. Ophthalmic Plast. Reconstr. Surg..

[15] De Simone M., Zoia C., Choucha A., Kong D.-S., De Maria L. (2024). The Transorbital Approach: A Comprehensive Review of Targets, Surgical Techniques, and Multiportal Variants. J. Clin. Med..

[16] De Simone M., Choucha A., Dannhoff G., Kong D.-S., Zoia C., Iaconetta G. (2024). Treating Trigeminal Schwannoma through a Transorbital Approach: A Systematic Review. J. Clin. Med..

[17] Rubin F., Pierrot S., Lebreton M., Contencin P., Couloigner V. (2013). Drainage of subperiosteal orbital abscesses complicating pediatric ethmoiditis: Comparison between external and transnasal approaches. Int. J. Pediatr. Otorhinolaryngol..

[18] Page E.L., Wiatrak B.J. (1996). Endoscopic vs External Drainage of Orbital Subperiosteal Abscess. Arch. Otolaryngol. –Head Neck Surg..

[19] Arjmand E.M., Lusk R.P., Muntz H.R. (1993). Pediatric sinusitis and subperiosteal orbital abscess formation: Diagnosis and treatment. Otolaryngol. –Head Neck Surg..

[20] Rahbar R., Robson C.D., Petersen R.A., DiCanzio J., Rosbe K.W., McGill T.J., Healy G.B. (2001). Management of Orbital Subperiosteal Abscess in Children. Arch. Otolaryngol.–Head Neck Surg..

[21] Kayhan F.T., Sayin I., Yazici Z.M., Erdur O. (2010). Management of orbital subperiosteal abscess. J. Craniofac Surg..

[22] Froehlich P., Pransky S.M., Fontaine P., Stearns G., Morgon A. (1997). Minimal Endoscopic Approach to Subperiosteal Orbital Abscess. Arch. Otolaryngol. –Head Neck Surg..

[23] Noordzij J.P., Harrison S.E., Mason J.C., Hashisaki G.T., Reibel J.F., Gross C.W. (2002). Pitfalls in the endoscopic drainage of subperiosteal orbital abscesses secondary to sinusitis. Am. J. Rhinol..

[24] Ketenci I., Unlü Y., Vural A., Doğan H., Sahin M.I., Tuncer E. (2013). Approaches to subperiosteal orbital abscesses. Eur. Arch. Oto-Rhino-Laryngol..

[25] Pereira K.D., Mitchell R.B., Younis R.T., Lazar R.H. (1997). Management of medial subperiosteal abscess of the orbit in children—A 5 year experience. Int. J. Pediatr. Otorhinolaryngol..

[26] Chorney S.R., Buzi A., Rizzi M.D. (2021). The Role of Endoscopic Sinus Surgery in Children Undergoing External Drainage of Non-Medial Subperiosteal Orbital Abscess. Am. J. Rhinol. Allergy.

[27] Pelton R.W., Smith M.E., Patel B.C.K., Kelly S.M. (2003). Cosmetic Considerations in Surgery for Orbital Subperiosteal Abscess in Children: Experience With a Combined Transcaruncular and Transnasal Endoscopic Approach. Arch. Otolaryngol. –Head Neck Surg..

[28] Gupta P., Saini M., Sharma V.K., Singh M., Abhaypal K., Mehta A. (2023). Postoperative Closed-Suction Drain in Anterior Orbitotomy. Cureus.

[29] Ikeda K., Oshima T., Suzuki H., Kikuchi T., Suzuki M., Kobayashi T. (2003). Surgical treatment of subperiosteal abscess of the orbit: Sendai’s ten-year experience. Auris Nasus Larynx.

